# In-situ preparation of sulfonated carbonaceous copper oxide-zirconia nanocomposite as a novel and recyclable solid acid catalyst for reduction of 4-nitrophenol

**DOI:** 10.1038/s41598-023-36627-x

**Published:** 2023-06-22

**Authors:** Mostafa Farrag

**Affiliations:** Chemistry Department, Faculty of Science, Assiut University, Assiut, 71515 Egypt

**Keywords:** Chemistry, Catalysis, Environmental chemistry, Green chemistry, Inorganic chemistry, Photochemistry, Physical chemistry

## Abstract

The missing-linker defects of UiO-66 were exploited to covalently anchor Cu nanoclusters (Cu/UiO-66). The molecular interactions between the metals and oxides as copper-zirconia interfaces in Cu/UiO-66 are essential for heterogeneous catalysis, leading to remarkable synergistic impacts on activity and selectivity. Homogeneously distributed carbonaceous mixed metal oxides (CuO/ZrO_2_@C) nanocomposite was prepared via carbonization of the Cu/UiO-66 at 600 °C for 3 h in air. To enhance the acidity properties of the CuO/ZrO_2_@C nanocomposite, a small amount of sulfuric acid was added and heated at 150 °C under an N_2_ atmosphere (CuO/ZrO_2_-SO_3_H@C). The synthesised Cu/UiO-66 and CuO/ZrO_2_-SO_3_H@C catalysts were used as novel catalysts in the reduction of 4-nitrophenol (4-NP) to 4-aminophenol (4-AP). The Cu/UiO-66 and CuO/ZrO_2_-SO_3_H@C catalysts displayed complete conversion of the 4-NP solution during (4 and 2 min) stirring at room temperature, respectively. These two catalysts exhibited a high reduction rate of 8.61 × 10^–3^ s^−1^, and 18.3 × 10^–3^ s^−1^, respectively. The X-ray photoelectron spectroscopic (XPS) analysis showed the charge of copper atoms in the Cu/UiO-66 catalyst was Cu^0^/Cu^II^ and in the CuO/ZrO_2_-SO_3_H@C catalyst was Cu^I^/Cu^II^ with nearly the same ratio (65/35). The particle size and the elemental composition of the CuO/ZrO_2_-SO_3_H@C catalyst were analysed by using high resolution transmission electron microscopy (HR-TEM), and energy-dispersive X-ray spectroscopy (EDS), and elemental mapping, respectively. The key point beyond the high catalytic activity and selectivity of the CuO/ZrO_2_-SO_3_H@C catalyst is both the carbon–metal oxides heterojunction structure that leads to good dispersion of the CuO and ZrO_2_ over the carbon sheets, and the high acidity properties that come from the combination between the Brønsted acid sites from sulfuric acid and Lewis acid sites from the UiO-66. The catalysts exhibited good recyclability efficiency without significant loss in activity, indicating their good potential for industrial applications.

## Introduction

At metal–metal oxide interfaces, phase boundaries produce distinct electronic structures as well as substrate binding, reactivity, and heterogeneous catalytic activity^1^. Cu nanoparticles supported on ZrO_2_ have attracted attention for their high activity and selectivity for the conversion of CO_2_ to methanol^2^, and hydrogen dissociation^2^. Recently, the atomically precise oxide node of a metal–organic framework (UiO-66) was used as a support for Cu clusters to ensure molecular-level proximity^1^. The UiO-66 with partly under-coordinated oxide nodes containing six Zr cations is used as a support for Cu clusters of varying nuclearity^3^. On the contrary, the conventional doping of Cu nanoparticles on ZrO_2_ support does not allow controlled variation of the interface and the metal nuclearity because of the inherent diversity of the oxide surface and the irregular porosity^1^. Due to the unique properties of MOFs such as a large surface area, tunable porosity, and a variety of structures, as well as good surface properties like high acidity and basicity. They can be used to make carbon nanomaterials, metal oxides, metal phosphides, metal chalcogenides, and metal carbides, among other nanostructured materials^4,5^.

Nitroaromatic compounds, such as nitrophenol derivatives, are critical intermediates in a variety of industries, medicines, paper manufacturing, fungicides, petrochemicals, pesticides, explosives, preservatives, insecticides, dyes, leather, and wood^6,7^. However, according to the U.S. Environmental Protection Agency (EPA), nitrophenol derivatives such as 4-nitrophenol (4-NP) are identified as a kind of organic pollutant and non-biodegradable pollutants^8^, moreover, the nitrophenol derivatives have toxic effects on the nervous system, viscera, and blood of human beings and animals^9^. To degrade nitrophenols, several methods have been developed, including the electro-Fenton method^10^, photocatalytic degradation^11^, and the electrochemical method^12^. The reduction of 4-nitrophenol (4-NP) to 4-aminophenol (4-AP) is an important reaction^13,14^, because 4-aminophenol is used to synthesise several important compounds such as drugs, rubber chemicals, and dyestuffs^15^.

Several catalysts were used to convert 4-NP to 4-AP, such as copper nanowires (Cu NWs)^16^, CuO@C dots^17^, M-BDC (M = Ag, Co, Cr, Mn, and Zr) metal–organic frameworks^18^, gold clusters (Au_25_) over Al_2_O_3_ and TiO_2_^13^, UiO-66/btb/Pd^19^ and Ag/UiO-66-NH_2_^20^. However, some of these catalysts are expensive and lack a high reduction rate. In our previous work, noble metals (Pt, Au, Pd, Ag) have been widely used in heterogeneous catalysis because of their physicochemical properties^21–30^. Nonetheless, due to the high cost of noble metals and their limited supply, there is a desire to use non-noble metals such as copper nanoparticles (Cu NPs), which have attracted considerable attention due to their cost effectiveness and stable nature^14,31^.

The electrophilic activation of a substrate by a Brønsted acid is undoubtedly the simplest and most common method for promoting numerous organic transformations^32–34^. Metal oxides are suitable catalyst supports in heterogeneous catalysis due to their interesting acid–base and redox properties, high chemical stability, commercial availability, and non-toxicity^35^. Carbon-based materials have also garnered interest as potential supports in heterogeneous catalysis. They are widely used in industry due to their unique properties, such as resistance to acidic and basic conditions, tunability of surface chemistry, good electric conductivity, and low cost^36^. Recently, it was discovered that dispersing carbon materials over inorganic supports results in organic/inorganic hybrid composites that have high stability, activity, and selectivity^13,37^.

The role of catalyst acidity in the wet peroxide oxidation of 4-nitrophenol (4-NP) and phenol was studied recently^38,39^. H_3_PO_4_, urea, and H_3_BO_3_ (P-, B-, and N-) doped carbon blacks (DCB) were prepared as catalysts for the reduction of 4-nitrophenol^38,39^. The conversion of 4-NP was 95.9, 65.5, and 40.4% over P-DCB, B-DCB, and N-DCB catalysts after 24 h of stirring at room temperature, where the acidity strengths were 1.33, 1.28, and 0.55 mmol g^−1^, respectively^38^. However, the pristine material (carbon black) reached only 6.8% conversion of 4-NP after the same reaction time^38^. Liu et al. confirmed that the hydrogenation of phenol to cyclohexanone was enhanced by increasing the acidity of the supported Pd-Lewis acid catalyst^40^.

In this context, for the first time, in-situ preparation of homogeneously distributed carbonaceous mixed metal oxides was achieved via carbonization of the covalently anchoring copper clusters in UiO-66 (Cu/UiO-66) at 600 °C, 3 h, and then sulfonated with sulfuric acid at 150 °C, 10 h under nitrogen atmosphere. The synergistic effect of the organic/inorganic hybrid materials and the Lewis acid and Brønsted acid sites as novel solid acid catalysts has appeared in the reduction of 4-nitrophenol to 4-aminophenol. Cu/UiO-66 and CuO/ZrO_2_-SO_3_H@C exhibited the highest catalytic activity with a rate of 8.61 × 10^–3^ s^−1^, and 18.3 × 10^–3^ s^−1^ at room temperature, respectively. The high catalytic activity and selectivity of the CuO/ZrO_2_-SO_3_H@C catalyst attributed to the homogenous dispersion of the CuO and ZrO_2_ over the carbon sheets, and the acidity properties that come from the Brønsted and Lewis acid sites. The effect of catalyst weight (50, 30, and 10 mg) was tested over the Cu/UiO-66 and sulfonated nanocomposites (CuO/ZrO_2_-SO_3_H@C, CuO-SO_3_H@C and ZrO_2_-SO_3_H@C) for reduction of 4-NP to 4-AP. The prepared catalysts showed high recyclability efficiency that is important for industrial applications. The particle size of the prepared catalysts was investigated by a high resolution transmission electron microscope (HR-TEM). The charge of the copper clusters was investigated by X-ray photoelectron spectroscopy (XPS). The crystallinity and surface texture properties of the prepared catalysts were measured by powder X-ray diffraction analysis and N_2_ gas sorption isotherm at − 196 °C, respectively. The acid strength and the number of acidic sites of the catalysts were measured by Orion 420 digital potentiometer.

## Experimental

### Chemicals

Zirconium chloride (ZrCl_4_), copper(II) nitrate trihydrate (Cu(NO_3_)_2_.3H_2_O, 98%), terephthalic acid (H_2_-BDC) linker, *N*,*N*-dimethylformamide (DMF), concentrated HCl and ethanol were purchased from Sigma–Aldrich to prepare the UiO-66 and Cu-BDC MOFs. Cupric acetate (CH_3_COO)_2_Cu·H_2_O, 98%) and acetone were purchased from Sigma–Aldrich to load the UiO-66 with copper ions (Cu/UiO-66). 4-nitrophenol (4-NP), 4-aminophenol (4-AP), and sodium borohydride (NaBH_4_, 96%, were purchased from Sigma-Aldrich to test the catalytic activity of the prepared catalysts. All chemicals were used as received. All glassware was thoroughly cleaned with aqua regia (HCl:HNO_3_ = 3:1 v/v), rinsed with twice distilled water and ethanol, and then dried in an oven before use.

### Synthesis of UiO-66

UiO-66 is synthesized as reported before by the Farha group^27^. Briefly, 1.25 g of ZrCl_4_ is suspended in 50 ml DMF and 10 ml conc. HCl is added dropwise, and then the solution is sonicated for 20 min until fully dissolved. 1.23 g of terephthalic acid (H_2_-BDC) linker is dissolved in 100 ml of DMF and added to the previous solution. The solution is sonicated for more than 20 min and then heated in an oven at 80 °C for 16–18 h. The resulting solid is separated by centrifuge (6000 rpm, 10 min) and washed first with DMF (2 × 30 ml) and then with EtOH (2 × 30 ml). Finally, UiO-66 is dried in an oven at 100 °C overnight. The MOF is activated at 150 °C, 12 h before measuring N_2_ isotherm^41^.

### Synthesis of Cu-BDC MOF

Cu-BDC was synthesized according to a published procedure^42,43^. In a typical preparation, 1.45 g Cu(NO_3_)_2_.3H_2_O (6 mmol), and 1 g terephthalic acid (6 mmol) were dissolved in 75 ml DMF (1:1 molar ratio). The resulting mixture was stirred for 10 min at room temperature and then heated at 110 °C for 36 h. The blue precipitate was obtained, filtrated, and washed with DMF several times, and then the MOF was dried in an oven at 220 °C for 24 h.

### Synthesis of Cu/UiO-66

Cu/UiO-66 was prepared according to a published procedure^1,5^. In a typical experiment, 0.5 g UiO-66 was suspended in 300 ml of an aqueous solution of copper acetate (0.01 M). The suspension was stirred for 24 h at room temperature. The pH of the solution was adjusted at 5–6 during the ion exchange of Cu acetate for which Cu(OH)^+^ at this pH was in contact with the OH groups on the Zr_6_ nodes^1,5^. After the ion exchange, the product was separated by centrifugation (6000 rpm, 10 min) and washed three times with twice distilled water. Then, the product was suspended in 50 ml acetone for 12 h to exchange the solvent. This step was repeated three times. The solvent-exchanged sample was then collected and dried at 120 °C in a vacuum oven for 12 h. The Cu^+2^ cations were reduced to Cu^0^ clusters by using 10 vol. % H_2_/N_2_ at 200 °C, for 3 h^1^.

### Preparation of carbonaceous metal oxide nanocomposites

To synthesize the carbonaceous ZrO_2_, CuO, and CuO/ZrO_2_ nanocomposites, the resulting metal–organic frameworks (UiO-66, Cu-BDC, and Cu/UiO-66) are placed in a muffle furnace at 600 °C for 3 h under air atmosphere using ramping temperature 10 °C/min^28^, donated as (ZrO_2_@C, CuO@C, and CuO/ZrO_2_@C, respectively).

### General procedure for the synthesis of sulfonated carbonaceous metal oxide nanocomposites

The carbonaceous metal oxides nanocomposites (ZrO_2_@C, CuO@C, and CuO/ZrO_2_@C) were sulfonated by heating in concentrated sulfuric acid (96–98 wt%) at 150 °C for 10 h under N_2_ atmosphere. The nanocomposite material obtained was then washed repeatedly with hot distilled water at 80 °C until sulfate anions were no longer detected in the filtered water. Sulfonated carbonaceous metal oxides nanocomposites were finally dried in an oven at 100 °C for 2 h^32^, donated as (ZrO_2_-SO_3_H@C, CuO-SO_3_H@C, and CuO/ZrO_2_-SO_3_H@C, respectively).

### Determination of the acidity of the synthesized solid acid catalysts

Potentiometric titration is used to investigate the acidity properties of the synthesized solid acid catalysts^44^. 0.1 g of each catalyst is suspended in acetonitrile for 2 h and then titrated with n-butylamine as a base in acetonitrile (0.025 N) at a rate of 0.1 ml over each 10 min. A double-junction electrode potentiometer (Orion 420 digital) is used to measure the potential.

### Catalytic activity of the synthesized catalysts

To investigate the catalytic activity of the synthesized catalysts, 50 ml of 4-NP (2 mM) was added to 50 mg of the catalysts, then 1.25 ml of NaBH_4_ (2 M) was added under constant stirring^13^. The yellow color of the solution gradually transformed to colorless, indicating the reduction of 4-NP into 4-AP, during the hydrogenation process. Several cuts were directly withdrawn from the reaction medium after a certain regular interval stirring time followed by measuring using a UV–Vis spectrophotometer. The decrease in intensity of the absorption peak of the 4-NP and NaBH_4_ mixture at 400 nm was followed up. The kinetic parameters of the reduction reaction were calculated. The effect of catalyst weight (50, 30, and 10 mg) was studied. At the end of the reaction, the catalyst was separated from the suspension by centrifugation (6000 rpm), washed several times with water and dried for 2 h at 110 °C, and then reused to study the recyclability of the prepared catalysts. The product 4-aminophenol was also identified by capillary column gas chromatography^13^.

## Results and discussion

We reported previously two methods to introduce metal nanoclusters in MOF pores^28,29^, where the metal nanoclusters were prepared by using protecting agents, such as l-cysteine^25,26^, l-glutathione^27,45^ and 2-phenylethyl thiol^29,46,47^. These clusters were loaded over or in the MOFs using the simple impregnation method^29^ or in-situ impregnation during the building steps of the MOFs^25^. Recently, naked copper clusters (25 ± 4 Cu atoms) were immersed in UiO-66 pores (Cu/UiO-66), where the missing-linker defects in the UiO-66 structure (~ 10%) were replaced by two -OH/OH_2_ species, which are active for ion exchange to deposit metal onto the Zr_6_ nodes of the MOF^1,48^. This method allows specific homotopic anchoring of naked metal clusters at the node^1,48^. According to the density functional theory (DFT) calculations, the average diameter of the copper clusters is 0.7–0.8 nm and the coordination numbers of Cu–O and Cu–Zr are 0.3–0.4, indicating that approximately 30% of the Cu atoms are bonded to the Zr_6_O_8_ nodes via Cu–O–Zr bridges^1^. This particle size can anchor inside the octahedral and tetrahedral cages of UiO-66 (1.3 and 0.9 nm, respectively)^1^. For the first time, in-situ preparation of sulfonated carbonaceous copper oxide-zirconia nanocomposite as novel and recyclable solid acid catalyst via carbonization of Cu/UiO-66 at 600 °C for 3 h under air atmosphere and then heating in concentrated sulfuric acid (96–98 wt%) at 150 °C for 10 h under N_2_ atmosphere.

### Catalysts characterization

**Figure 1:**
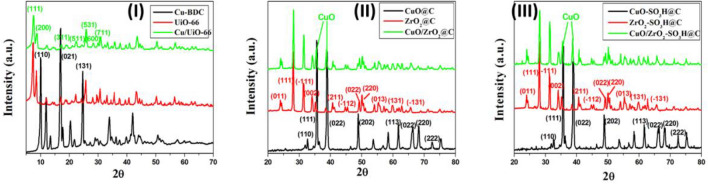
X-ray diffractograms of (**I**) bare Cu-BDC and UiO-66 and copper clusters inside the missing-linker defects of UiO-66, (**II**) carbonaceous metal oxides (CuO@C, ZrO_2_@C, and CuO/ZrO_2_@C) nanocomposites, (**III**) the sulfonated metal oxide@carbon (CuO-SO_3_H@C, ZrO_2_-SO_3_H@C, and CuO/ZrO_2_-SO_3_H@C) nanocomposites.

The crystallinity of the prepared catalysts was confirmed using X-ray diffraction (XRD). Figure 1-I represents the XRD pattern of the parent Cu-BDC with high intensity diffraction peaks at (2θ) 10.04°, 17.02°, and 24.70°, corresponding to the (110), (021), and (131) planes, respectively^49^, which are indicating a high crystallinity degree of Cu-BDC^43,49^. The XRD pattern of the UiO-66 shows characteristic peaks at 2θ = 7.3°, 8.5°, and 25.7°, corresponding to the (111), (200), and (531) planes, respectively (Fig. 1-I), which are well matched with the simulated XRD pattern of UiO-66^50^. The XRD pattern of the as-synthesized Cu/UiO-66 is in excellent agreement with the XRD pattern for the parent UiO-66^28,29^, which confirms the high dispersion and lower loading percentage of the Cu nanoclusters inside the MOF's pores (Fig. 1-I)^28^.

The prepared MOFs (Cu-BDC and UiO-66) were calcined at 600 °C in the air to prepare the carbonaceous CuO and ZrO_2_ nanocomposites, respectively. The crystallinity of the nanocomposites was investigated by XRD (Fig. 1-II). The XRD diffraction peaks of CuO@C are sharp and strong, indicating that the sample is high crystalline quality and has a monoclinic structure with lattice parameters a = 0.4685 nm, b = 0.3425 nm, and c = 0.5130 nm, which is in good agreement with JCPDS card number 45-0937^51^. The diffraction peaks of the CuO@C at 2θ of 32.6°, 35.8°, 39°, 48.86°, 58.3°, 61.68°, 66.41°, 68.26° and 72.6° could be ascribed to characteristic reflections from (110), (111), (022), (202), (202), (113), (022), (220) and (222) planes, respectively (Fig. 1-II). The XRD pattern of the ZrO_2_@C shows crystalline diffraction peaks at 2θ of 24.1°, 28.2°, 31.5°, 34.2°, 40.6°, 44.74°, 49.29°, 50.1°, 55.54°, 59.8° and 65.84° that correspond to (011), (111), (− 111), (002), (211), (− 112), (022), (220), (013), (131) and (− 131) planes, respectively, that are simulated with the monoclinic ZrO_2_ (ICDD File No. 37–1484)^52^. The XRD pattern of the carbonaceous Cu/UiO-66 showed the same XRD pattern for ZrO_2_@C plus two diffraction peaks at 35.8° and 39° indicating the presence of CuO in the CuO/ZrO_2_@C nanocomposites (Fig. 1-II).

The prepared nanocomposites were treated with sulfuric acid at 150 °C under an N_2_ atmosphere to prepare sulfonated metal oxide@carbon nanocomposites with Brønsted acid sites. The sulfonation process does not affect the crystallinity of the carbonaceous metal oxides. Figure 1-III shows the XRD diffraction patterns of the CuO-SO_3_H@C, ZrO_2_-SO_3_H@C, and CuO/ZrO_2_-SO_3_H@C nanocomposites which are similar to the carbonaceous metal oxides (Fig. 1-II).

**Figure 2:**
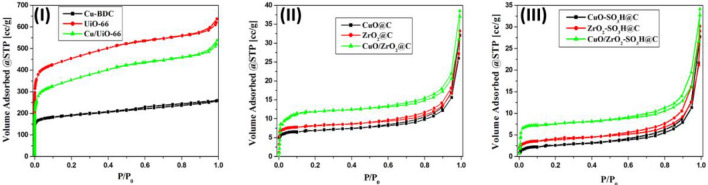
The nitrogen adsorption–desorption isotherms of (**I**) bare Cu-BDC and UiO-66 and copper clusters inside the missing-linker defects of UiO-66, (**II**) carbonaceous metal oxides (CuO@C, ZrO_2_@C and CuO/ZrO_2_@C) nanocomposites, (**III**) the sulfonated metal oxide@carbon (CuO-SO_3_H@C, ZrO_2_-SO_3_H@C, and CuO/ZrO_2_-SO_3_H@C) nanocomposites.

The N_2_ adsorption–desorption isotherms at − 196 °C were used to investigate the textural properties of the prepared catalysts, as shown in Fig. 2 and summarised in Table 1. The Brunauer − Emmett − Teller (BET) equation was used to measure the specific surface areas of the prepared MOFs (Cu-BDC and UiO-66) 644 and 1315 m^2^/g, respectively, (Fig. 2-I). The S_BET_ of the Cu/UiO-66 decreases slightly due to the partial occupation of the UiO-66 pores with the deposited Cu nanoclusters (Table 1). The parent MOFs and loaded UiO-66 with copper clusters exhibit type I adsorption–desorption isotherms with H4 hysteresis loops according to the IUPAC classification of hysteresis loops^53^. The specific surface areas of the prepared MOFs are far higher than the carbonaceous copper and zirconium oxide (Fig. 2-II) and sulfonated carbonaceous copper and zirconium oxide (Fig. 2-III), as summarised in Table 1. The specific surface area of the CuO/ZrO_2_@C nanocomposite is higher than the carbonaceous copper and zirconium oxide due to the contribution of the copper species^1^.

**Table 1:** Surface area and pore volume data of the prepared catalysts**.**

Catalysts	S_BET_ (m^2^ g^−1^)	S_t_ (m^2^ g^−1^)	Pore volume (cm^3^/g) × 10^–3^
Cu-BDC	644	644	420
UiO-66	1315	1314	650
Cu/UiO-66	1220	1221	620
CuO@C	21	21	77
ZrO_2_@C	47	46	140
CuO/ZrO_2_@C	65	65	170
CuO-SO_3_H@C	18	18	59
ZrO_2_-SO_3_H@C	43	44	122
CuO/ZrO_2_-SO_3_H@C	61	60	156

The Barrett-Joyner-Halenda (BJH) method is used to measure the pore size distribution of the prepared catalysts (Table 1). The total pore volume of Cu-BDC and UiO-66 is 420 × 10^–3^ and 650 × 10^–3^ cm^3^/g, respectively. The total pore volume of the Cu/UiO-66 catalyst decreases slightly due to the incorporation of the Cu clusters inside the UiO-66 pores (Table 1). However, there is a considerable variation in the pore volume distribution of the parent and loaded MOFs and the carbonaceous CuO and ZrO_2_ nanocomposites, where the pore volume of CuO@C, ZrO_2_@C, and CuO/ZrO_2_@C nanocomposites are 77 × 10^–3^, 140 × 10^–3^ and 170 × 10^–3^ cm^3^/g (Table 1), respectively. The specific surface areas of the prepared catalysts are measured by another method, the T-method (S_t_) which shows the same values as S_BET_ (Table1), which confirms the correct choice of the standard t-curves for pore analysis^22,24,28^.

**Figure 3:**
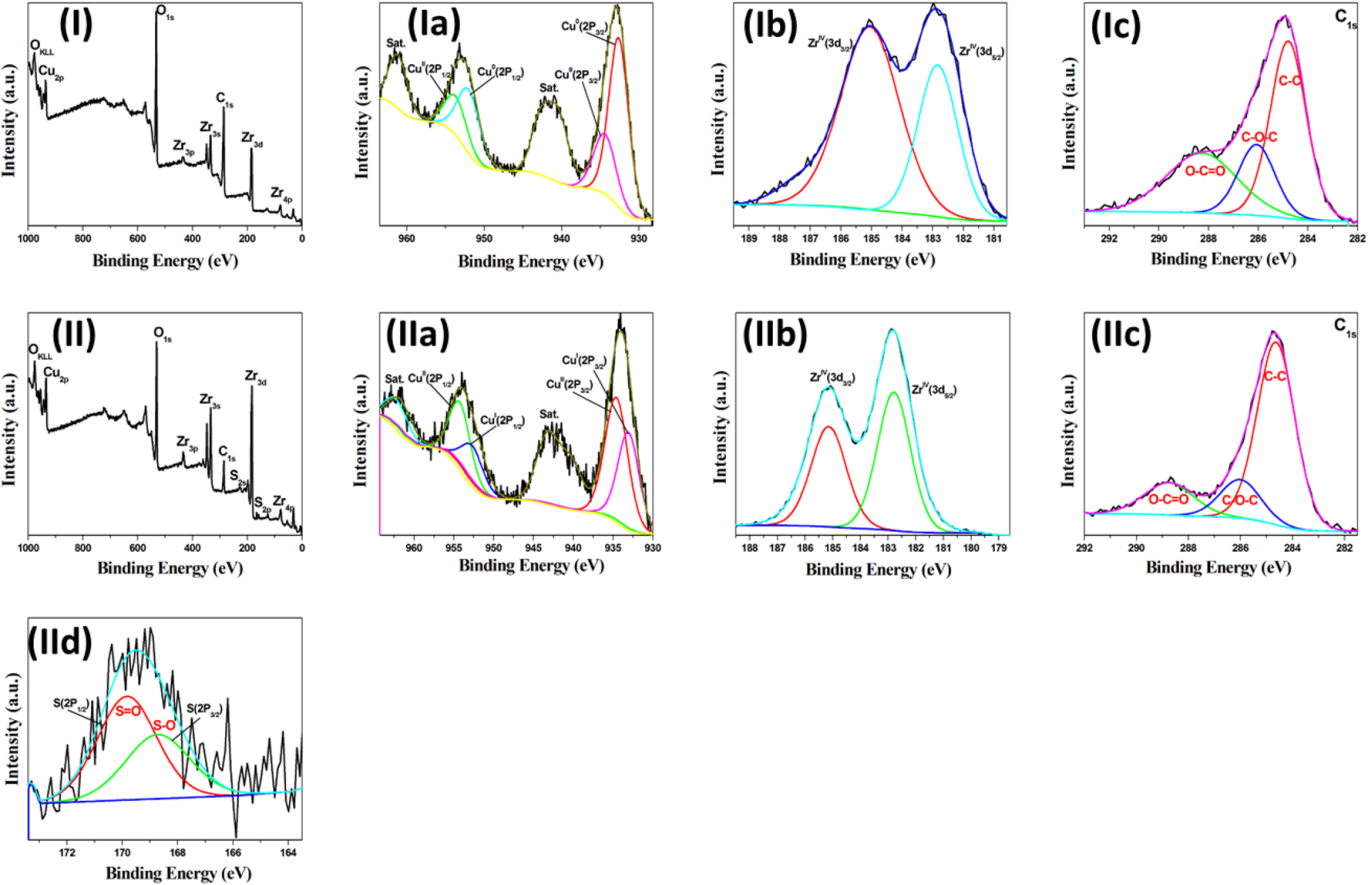
HR-XPS spectra of the Cu/UiO-66 and CuO/ZrO_2_-SO_3_H@C catalysts, (**I**) survey spectrum of Cu/UiO-66, (**Ia**) HR-XPS spectrum of Cu 2p, (**Ib**) HR-XPS spectrum of Zr 3d, and (**Ic**) HR-XPS spectrum of C 1s. (**II**) Survey spectrum of CuO/ZrO_2_-SO_3_H@C, (**IIa**) HR-XPS spectrum of Cu 2p, (**IIb**) HR-XPS spectrum of Zr 3d, (**IIc**) HR-XPS spectrum of C 1s and (**IId**) HR-XPS spectrum of S 2p.

The X-ray photoelectron spectroscopy (XPS) technique is used to demonstrate the chemical composition and the charge state of the Cu nanoclusters in the prepared nanocomposites^28–31^. Figure 3-I displays the survey XPS spectrum of the Cu/UiO-66 that indicates the catalyst contains four elements Zr 3d, 4p, 3s and 3p, Cu 2p, C 1s, and O 1s. The high resolution Cu 2p XPS spectrum was shown in Fig. 3-Ia, the two peaks at binding energies 932.6 and 934.4 eV that corresponding to Cu 3p_3/2_ were related to Cu (0) and Cu (II), respectively^31,54^. Another two peaks appear at binding energies of 952.5 and 954.3 eV, which correspond to Cu 3p_1/2_ and related to Cu (0) and Cu (II), respectively^54^. The peak position and the corresponding satellites indicate exists of CuO (Fig. 3-Ia). The satellite peaks originate from numerous excitations in copper oxides^54^. The Cu^0^/Cu^II^ ratio in the Cu/UiO-66 catalyst is 65/35%, which is in good agreement with the X-ray absorption near edge structure (XANES) technique^1^. Figure 3-Ib displays the Zr 3d_5/2_ and 3d_3/2_ peaks, which are observed at 182.8 eV and 185.1 eV, respectively, and are in good agreement with the published XPS spectrum for ZrO_2_^55^. These peaks strongly imply that Zr^4+^ is the most common oxidation state for Zr^54^. The deconvoluted C1s peak can be fitted into three peaks at binding energy values of 284.6, 286, and 288.4 eV referring to C–C, C–O–C, O–C=O, respectively (Fig. 3-Ic)^30,56^.

**Figure 4:**
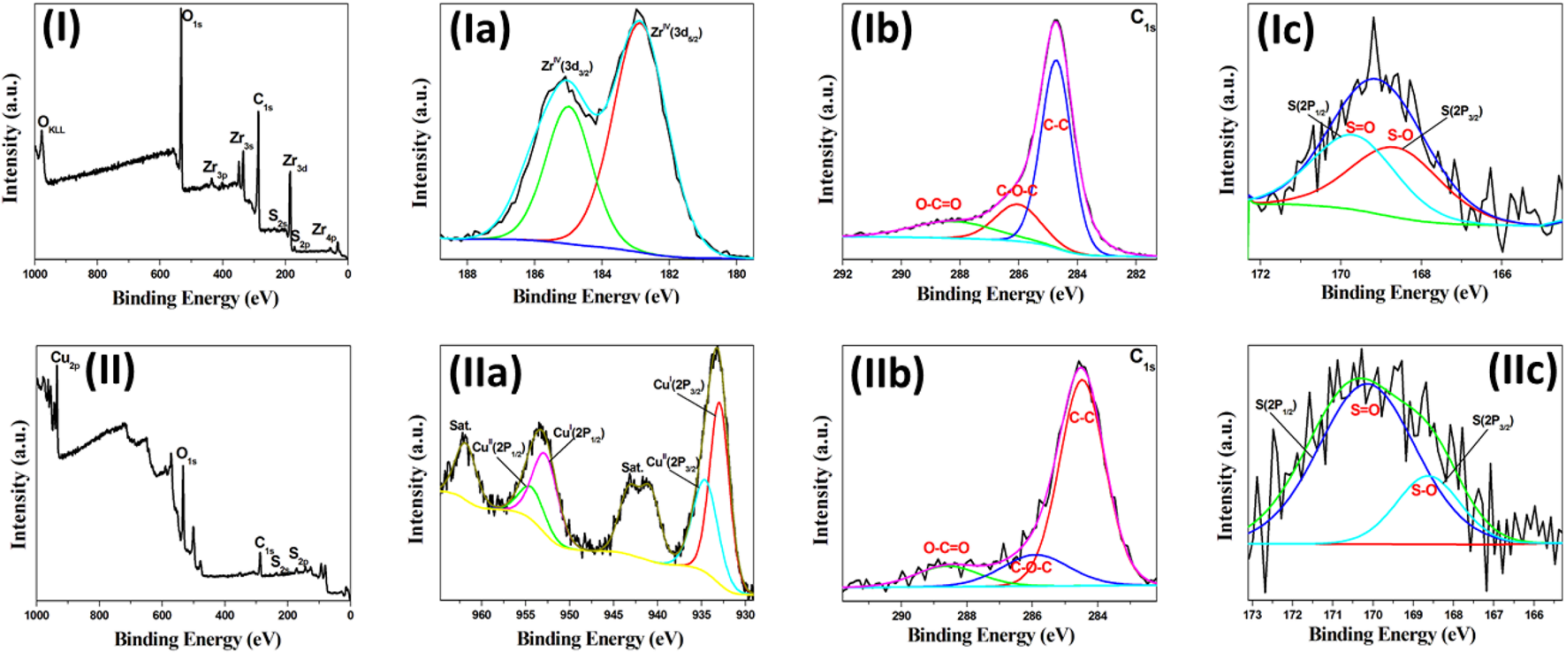
HR-XPS spectra of the ZrO_2_-SO_3_H@C and CuO-SO_3_H@C catalysts, (**I**) survey spectrum of ZrO_2_-SO_3_H@C, (**Ia**) HR-XPS spectrum of Zr 3d, and (**Ib**) HR-XPS spectrum of C 1s and (**IIc**) HR-XPS spectrum of S 2p. (**II**) Survey spectrum of CuO-SO_3_H@C, (**IIa**) HR-XPS spectrum of Cu 2p, (**IIb**) HR-XPS spectrum of C 1s, and (**IIc**) HR-XPS spectrum of S 2p.

XPS analysis is used to identify the chemical structure of the prepared sulfonated carbonaceous metal oxides (CuO/ZrO_2_-SO_3_H@C, ZrO_2_-SO_3_H@C, and CuO-SO_3_H@C). Figure 3-II shows the survey XPS spectrum of CuO/ZrO_2_-SO_3_H@C that indicates the catalyst contains five elements Zr 3d, 4p, 3s and 3p, Cu 2p, C 1s, O 1s, and S 2p. The high resolution Cu 2p XPS spectrum confirms the CuO/ZrO_2_-SO_3_H@C has a mixture of two copper oxides (Cu_2_O and CuO) as shown in Fig. 3-IIa. The two peaks at binding energies 933 and 953 eV that corresponding to Cu 3p_3/2_ and 3p_1/2_ are related to Cu (I), respectively^31,54^, and another two peaks at binding energies of 934.5 and 954.5 eV, that corresponding to Cu 3p_3/2_ and Cu 3p_1/2_ are related to Cu (II), respectively^54^. The Cu^I^/Cu^II^ ratio in the CuO/ZrO_2_-SO_3_H@C catalyst is 64/36% (Fig. 3-IIa). The high resolution XPS spectra of the Zr 2p and C 1s (Fig. 3-IIb,c) are the same as the above-mentioned peaks position of the Cu/UiO-66 catalyst, respectively. The S 2p spectrum of the CuO/ZrO_2_-SO_3_H@C catalyst shows two different peaks at binding energy 168.7 and 169.9 eV that are attributed to the S–O and S=O bonds (Fig. 3-IId). The peak separation of 1.2 eV that indicates the S in the CuO/ZrO_2_-SO_3_H@C is mainly in the form of SO_3_H groups bonded to the CuO/ZrO_2_ nanocomposite^57^. Figure 4-I shows the survey XPS spectrum of ZrO_2_-SO_3_H@C that indicates the catalyst contains four elements Zr 3d, 4p, 3s and 3p, C 1s, O 1s, and S 2p. The survey XPS spectrum of CuO-SO_3_H@C exhibits also four elements Cu 2p, C 1s, O 1s, and S 2p (Fig. 4-II). The high resolution XPS spectra of these elements are as mentioned above (Fig. 4).

**Figure 5:**
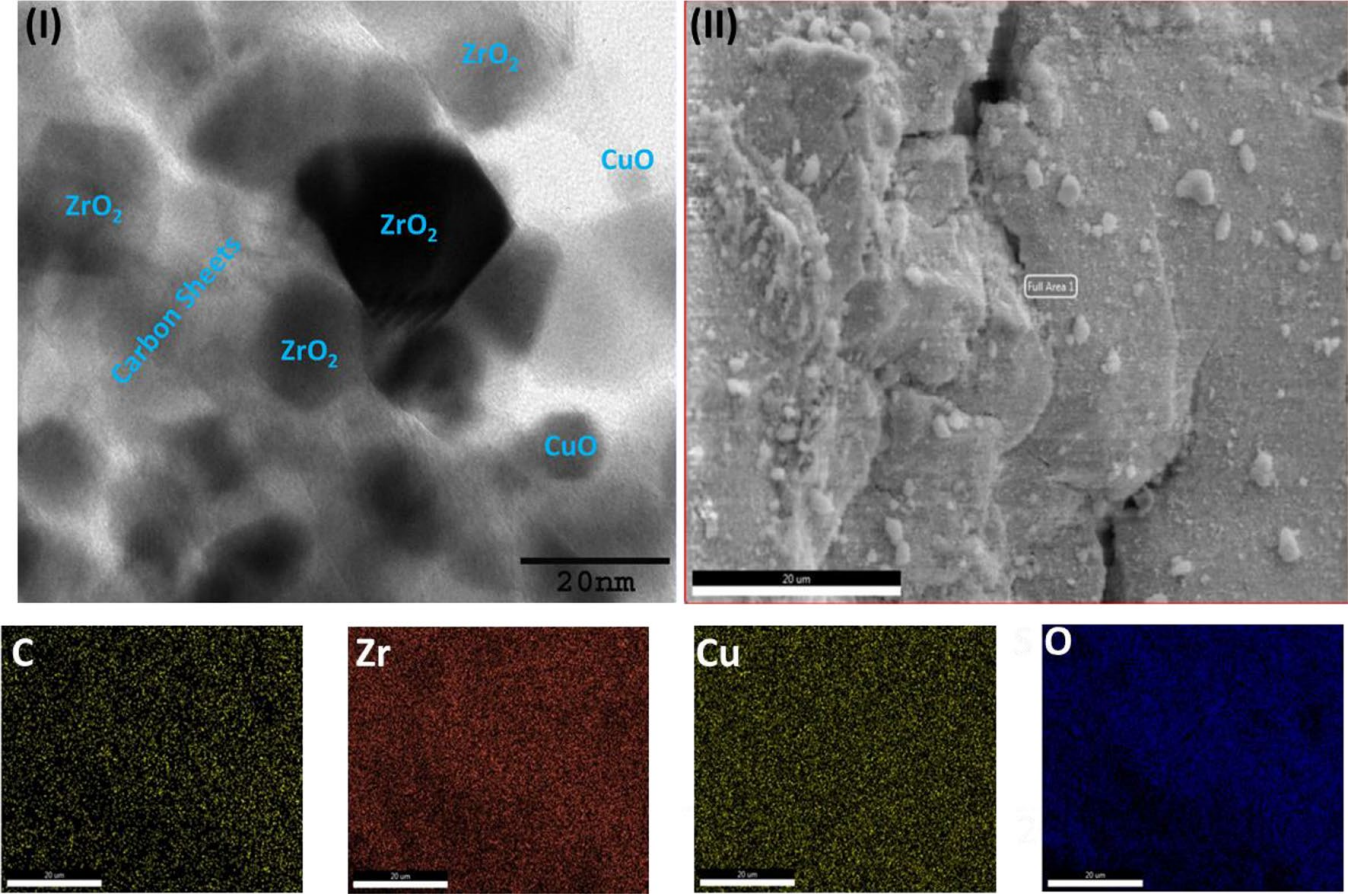
(**I**) HR-TEM image and (**II**) EDS and elemental mapping analysis of the CuO/ZrO_2_-SO_3_H@C.

The HR-TEM image of the CuO/ZrO_2_-SO_3_H@C (Fig. 5-I) displays the monoclinic structure of ZrO_2_ and CuO particles as confirmed by XRD analysis (Fig. 1-III). The particle size of the ZrO_2_ and CuO particles over the carbon sheets are 12–25 nm and 6–10 nm, respectively (Fig. 5-I). Figure 5-II exhibits the energy dispersive X-ray spectroscopy (EDS) and elemental mapping of the prepared CuO/ZrO_2_-SO_3_H@C to determine its elemental composition. The deeper investigation of the EDS data reveals that the C, Zr, Cu, O, and S elementals were unevenly present in the CuO/ZrO_2_-SO_3_H@C, as shown in Fig. 5-II. The random distribution of these elementals (C, Zr, Cu, and S) was strongly supported from the elemental mapping (specified by different colors) of the prepared CuO/ZrO_2_-SO_3_H@C (Fig. 5)^58^.

**Figure 6:**
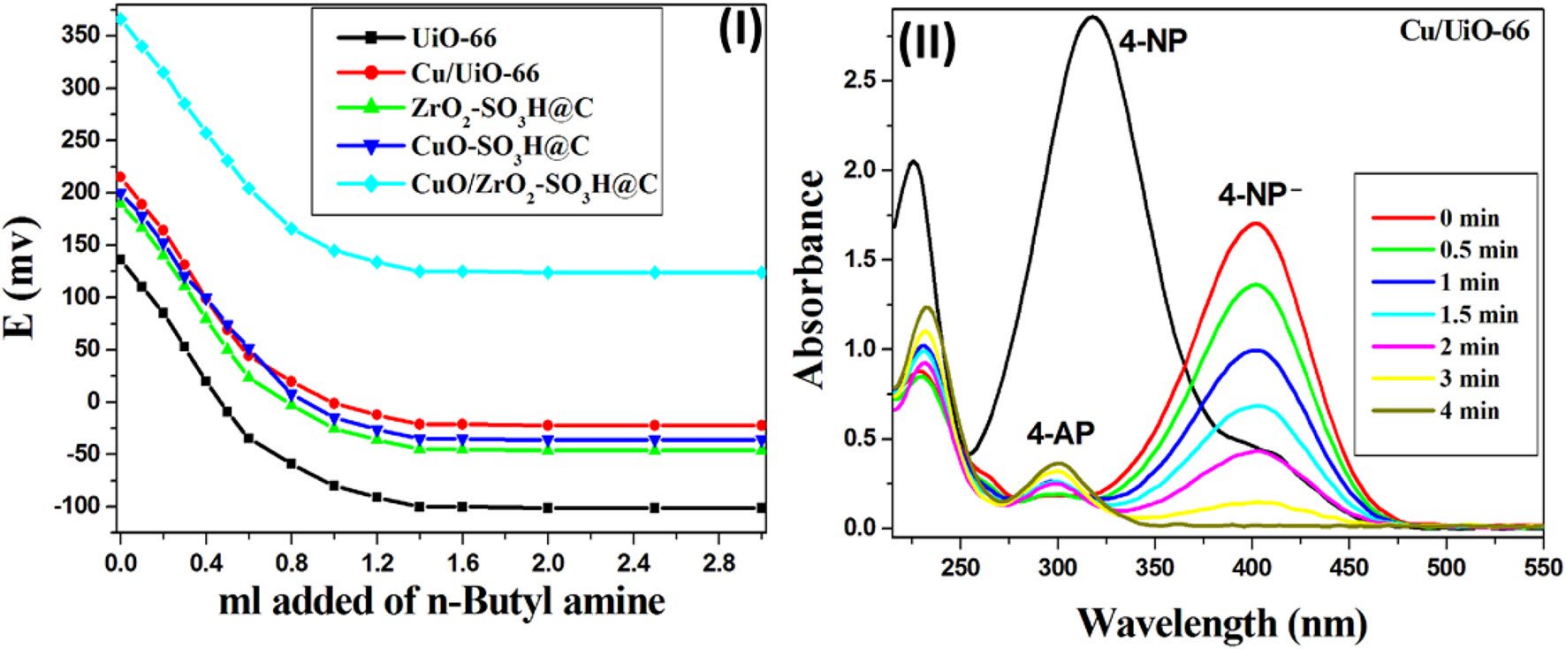
**(I)** Potentiometric titration curves of the prepared catalysts. (**II**) UV–Vis absorption spectra of 4-NP reduction reaction over Cu/UiO-66. The reaction conditions are 50 ml of 4-NP (2 mM), 50 mg of the catalyst, 1.25 ml of NaBH_4_ (2 M), and 1000 rpm.

The surface acidity of the prepared catalysts was determined using non-aqueous potentiometric titration by measuring the electrode potential variation by Orion 420 digital model. A known amount (0.1 g) of the prepared catalysts was suspended in acetonitrile for 2 h and then titrated with 0.025 N n-butylamine and the electrode potential was measured. The acid strength of the surface sites is determined by the electrode's initial potential (Ei) and the total number of acid sites are calculated from the curve plateau with mequiv/g units. The strength of the acid sites can be classified according to the following scale: Ei > 100 mV (very strong sites); 0 < Ei < 100 mV (strong sites); − 100 < Ei < 0 mV (weak sites); and Ei <  − 100 mV (very weak sites)^59^. The potentiometric titration curves are presented in Fig. 6-I, which illustrates the electrode potential variation versus volume added from *n*-butyl amine. The initial potential (Ei) of the neat UiO-66 and Cu-BDC equals 135.6 and 100 mV, respectively, which indicates they have moderate acidity^44,60^. The binding energy of NH_3_ in the undefective and defective regions of the UiO-66 is 75.8 and 110.1 kJ mol^−1^ per NH_3_ molecule, respectively, clearly demonstrating enhanced binding at the defect center^61^. The copper clusters were bonded in the defective region to form the Cu/UiO-66, where the Cu atoms formed the Cu–O–Zr bonds that have a significant formal positive charge, therefore enhancing the Lewis acidic properties of the UiO-66^1^. Incorporation of the copper clusters inside the UiO-66 frameworks increased the acid strength of the Cu/UiO-66 (Ei = 215 mV) and created strong acid sites on the surface, where the total number of acid sites is equal to 2.3 × 10^20^ mequiv g^−1^. Table 2 displays an increase in the total number of acid sites on the sulfonated carbonaceous metal oxides surface in this order CuO/ZrO_2_-SO_3_H@C > CuO-SO_3_H@C > ZrO_2_-SO_3_H@C. So, the treatment of the carbonaceous metal oxides with H_2_SO_4_ enhances their acidity properties. Moreover, the high acidity value of CuO/ZrO_2_-SO_3_H@C may be due to the synergetic effect between copper and zirconium metals. The total number of acid sites/g of the prepared catalysts was calculated from Eq. (1).

1 $${\text{Total}}\;{\text{number}}\;{\text{of}}\;{\text{acid}}\;{\text{sites}}/{\text{g}} = \left( {{\text{steady}}\;{\text{point}}\;{\text{of}}\;{\text{plateau}} * \left( {{\text{equiv}}./{\text{g}}} \right)*{\text{N}}_{{\text{A}}} } \right)/{1}00$$

**Table 2:** Acidity values of the prepared catalysts**.**

Catalyst	Number of acidic sites [mequiv g^−1^] × 10^20^	Acid strength (E_i_) [mV]
Cu-BDC	1.07	100
UiO-66	1.31	136.2
Cu/UiO-66	2.3	215
CuO@C	0.89	87
ZrO_2_@C	0.98	95
CuO/ZrO_2_@C	1.63	162
ZrO_2_-SO_3_H@C	2	190
CuO-SO_3_H@C	2.1	200
CuO/ZrO_2_-SO_3_H@C	3.05	366

### Catalytic activity of the synthesized catalysts

To examine the catalytic activity of the synthesized catalysts, the reduction of 4-NP to 4-AP using NaBH_4_ as a reducing agent was used as a model test reaction^13^. The change in absorbance of the 4-nitrophenolate peak at 400 nm was used to calculate the degree of reduction of 4-NP to 4-AP. The decrease in absorption intensity at 400 nm for 4-NP^−^ and the increase in absorption intensity at 300 and 230 nm for 4-AP, indicate the reduction of 4-nitrophenol (Fig. 6-II). Whereas the standard electrochemical potential of 4-NP equals (E_o_ (4-NP/4-AP) =  − 0.76 V), and NaBH_4_ equals (E_o_ (H_3_BO_3_/BH_4_^−^) =  − 1.33 V), the reduction process is thermodynamically favourable. However, it is kinetically unfavourable due to the mutually repelling negative ions such as NP^−^ and BH_4_^−^^62^.

**Figure 7:**
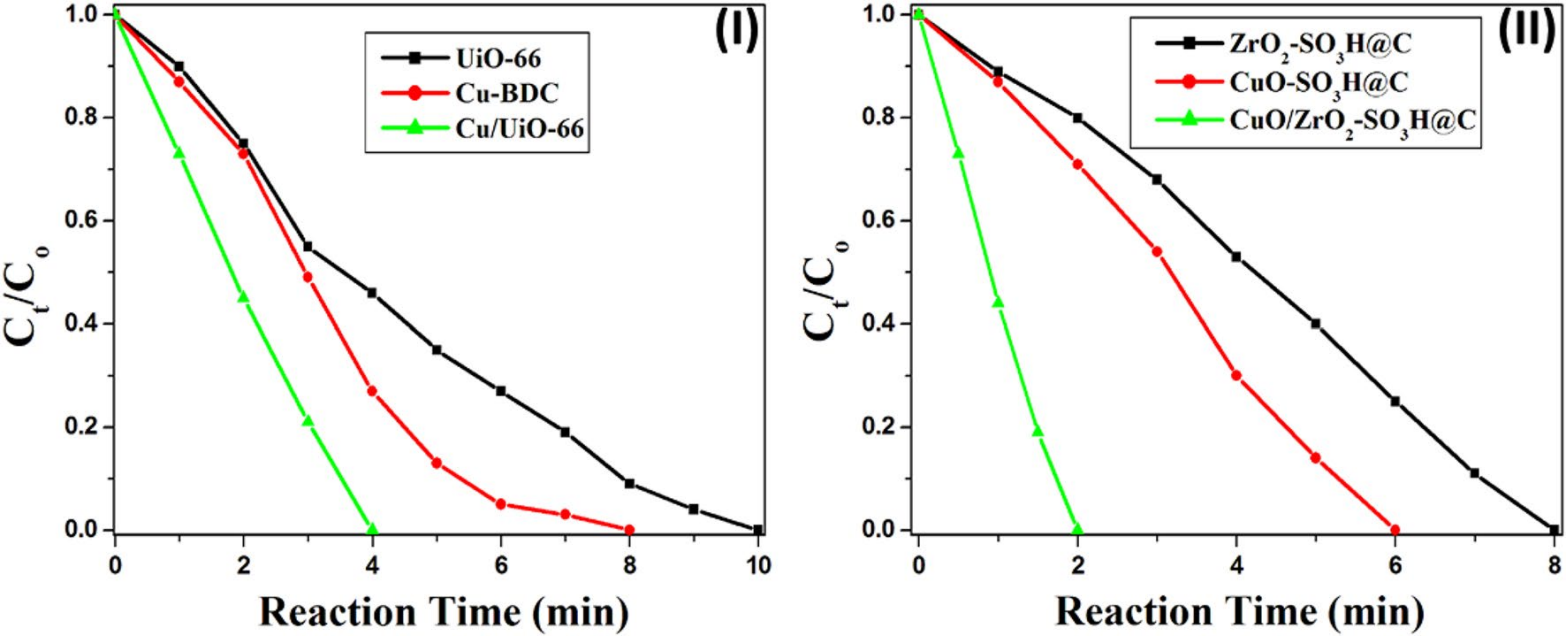
**(I–II)** The change in the concentration of 4-NP with time during the reduction reaction over the prepared catalysts, (the reaction conditions are 50 ml of 4-NP (2 mM), 50 mg of the catalyst, 1.25 ml of NaBH_4_ (2 M), and 1000 rpm).

Figure 6-II shows the UV–Vis spectra of 100% conversion of 4-NP solution into 4-AP within only 4 min of stirring over Cu/UiO-66 at room temperature, while UiO-66 and Cu-BDC require 10 and 8 min for full reduction of 4-NP, respectively (Fig. 7-I). Rath et al. confirmed that metallic Cu is a far superior catalyst for the conversion of 4-NP to 4-AP when compared to Cu_2_O and CuO^63^. In our case, the unique atomic packing structure and electronic properties of the copper clusters (25 ± 4 Cu atoms) are responsible for their extraordinary catalytic activity in the reduction of 4-nitrophenol. The acidic properties of the UiO-66 were enhanced by the incorporation of the Cu clusters, where the Cu atoms form the Cu–O–Zr bonds that have a significant formal positive charge^1^. Whereas the total number of acid sites in the UiO-66 increased from 1.31 × 10^20^ to 2.3 × 10^20^ mequiv g^−1^ by incorporation of the copper clusters (Cu/UiO-66)^64^.

Filiz studied the effect of support material on the catalytic reduction of 4-NP using CuO nanoparticles, the supports were ordered as follows: ZrO_2_ > Al_2_O_3_ > SiO_2_ > CaO > MgO > ZnO^65^. In this work, we prepared a carbonised CuO/ZrO_2_@C nanocomposite via calcination of Cu/UiO-66 at 600 °C in air. CuO/ZrO_2_@C shows high catalytic activity in the complete conversion of 4-NP into 4-AP within only 10 min of stirring in comparison to CuO@C and ZrO_2_@C (Fig. S1). The carbon sheets of CuO/ZrO_2_@C nanocomposite provide efficient adsorption of 4-NP due to the functional groups of carbon, such as non-covalent interactions including π-π stacking, hydrogen bonds, and so on^62^.

Recently, Mhlwatika et al. prepared a series of perovskite materials (ABO_3_) as heterogeneous catalysts in the reduction of 4-nitrophenol, the activity of these catalysts does not depend on the surface area but depends on the acidic strength^66^. UiO-66, MOF-5 (Zn-BDC), and MIL-101 (Fe-BDC) were used to activate the reduction of 4-nitrophenol to 4-aminophenol, the result indicates that UiO-66 exhibited the best catalytic behavior due to its Lewis acidic nature at the metal nodes^64^. Moreover, many MOFs were used as catalysts in different reactions due to their unique properties^67–69^. Therefore, we planned to enhance the catalytic activity of the carbonaceous metal oxides by treating them with sulfuric acid under an N_2_ atmosphere. Sulfonated carbonaceous metal oxides (ZrO_2_-SO_3_H@C, CuO-SO_3_H@C, and CuO/ZrO_2_-SO_3_H@C) exhibited amazing catalytic activity in the reduction of 4-nitrophenol (Fig. 7-III). The CuO/ZrO_2_-SO_3_H@C catalyst succeeded in the reduction of the 4-nitrophenol solution into 4-aminophenol within only 2 min of stirring at room temperature (Fig. 7-II). The catalyst has very strong acid sites according to the classification (Ei > 100 mV)^59^, where its initial potential (Ei) is equal to 366 mV and the total number of acidic sites is equal to 3.05 × 10^20^ mequiv g^−1^ (Table 2). The reason for the effect of the acid sites is still under investigation. However, we expect that the acid sites permit better adsorption of nitrophenol on the catalyst surface and promote the breakage of the N–O bond in the intermediate phenylhydroxylamine, thus facilitating the reactions. Furthermore, the generated protons from the hydrolysis of NaBH_4_ do not strongly adsorb on the acidic surface, they are readily available to participate in the conversion of 4-NP^66,70^.

**Figure 8:**
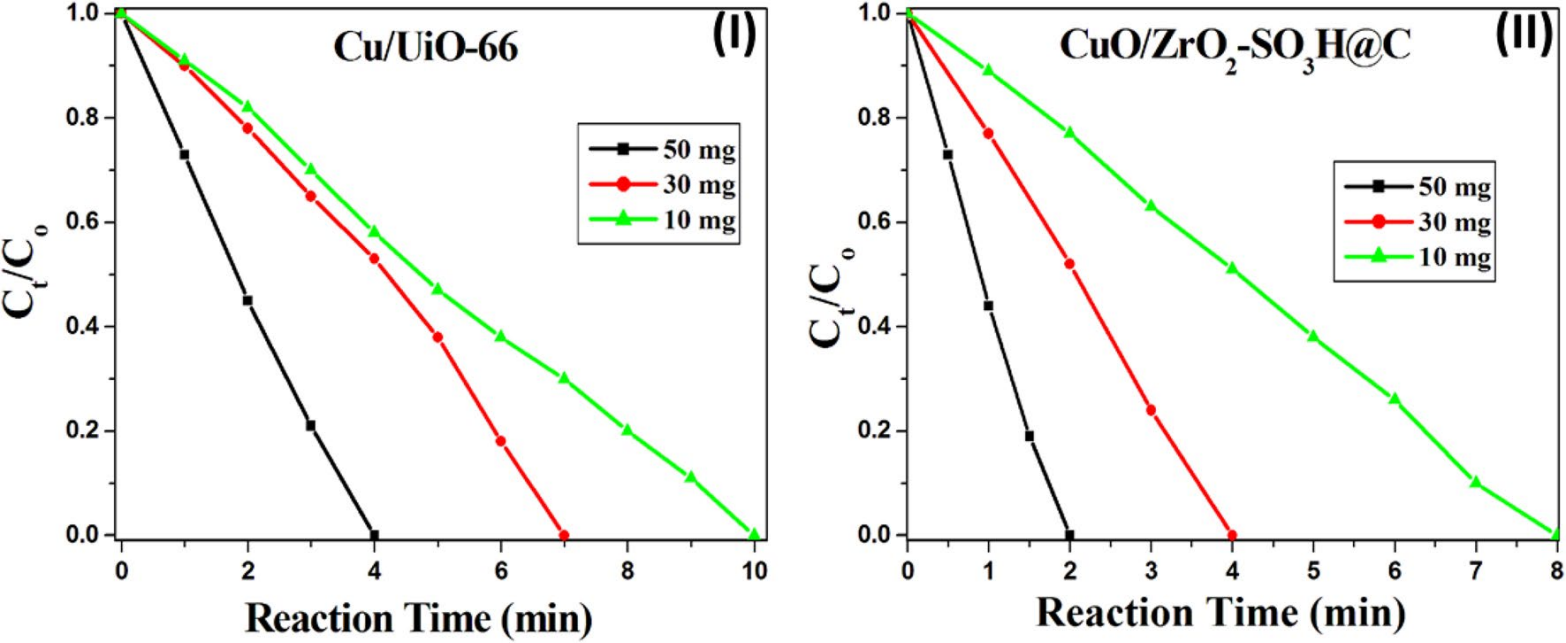
The effect of catalyst weight on the reduction of 4-NP over the Cu/UiO-66 (**I**), and CuO/ZrO_2_-SO_3_H@C (**II**). The experimental conditions were kept constant, but the catalyst weight was changed only (50, 30, and 10 mg).

The effect of catalyst weight (50, 30, and 10 mg) on the reduction of 4-NP was investigated (Fig. 8 and Fig. S2). The time of the reaction was increased with the decrease of the catalyst loading. The time to complete conversion of 4-NP is 2 min, 4 min, and 8 min over 50 mg, 30 mg, and 10 mg of the CuO/ZrO_2_-SO_3_H@C, respectively (Fig. 8-II).

**Figure 9:**
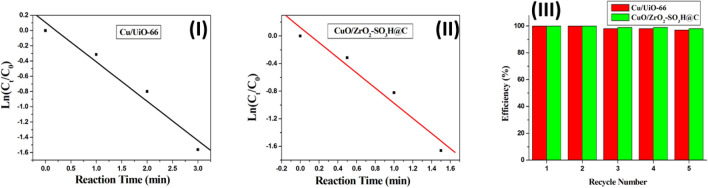
The kinetic plots of Cu/UiO-66 (**I**) and CuO/ZrO_2_-SO_3_H@C (**II**). (**III**) Recyclability effect of Cu/UiO-66 and CuO/ZrO_2_-SO_3_H@C catalysts. (The reaction conditions are 50 ml of 4-NP (2 mM), 50 mg of the catalyst, 1.25 ml of NaBH_4_ (2 M), and 1000 rpm).

We chose the Cu/UiO-66 and CuO/ZrO_2_-SO_3_H@C catalysts to calculate the rate constant of the reduction reaction using the equation ln(C_t_/C_0_) = ln(A_t_/A_0_) =  − kt, where C_t_ is the concentration of 4-NP at time t, C_0_ is the initial concentration, and k is the apparent rate constant (Fig. 9). As a result, the reaction process can be described as a pseudo-first-order reaction in terms of 4-NP concentration. The constant rates of Cu/UiO-66 and CuO/ZrO_2_-SO_3_H@C are 8.61 × 10^–3^ s^−1^ and 18.3 × 10^–3^ s^−1^, respectively (Fig. 9-I–II). The two catalysts were also used to study reusability (Fig. 9-III). The reusability of Cu/UiO-66 and CuO/ZrO_2_-SO_3_H@C has been investigated several times (Fig. 9-III). The catalysts can be reused up to five times without losing their catalytic performance^71^. The catalytic efficiency of Cu/UiO-66 and CuO/ZrO_2_-SO_3_H@C catalysts is nearly 100% without significant loss in activity during the five cycles (Fig. 9-III).

The mechanism for the reduction of 4-NP into 4-AP is presented in Fig. S3. Firstly, the sodium borohydride produces hydrogen, which is adsorbed on the catalyst surface, and then the reduction process happens. The reduction process requires six protons (6H^+^) to convert the nitro group (NO_2_) to an amino group (NH_2_).

### Catalytic activity comparison

Table 3 shows a comparison between our prepared catalysts and other reported catalysts. Different families of catalysts were summarized in this table, some of them carbonized metal oxides such as Cu_x_O@C^72^ and CoO_x_/CN^73^, doped MOFs, graphene oxide, and dendrimers with noble metals (Pd and Ag)^74–76^ and mixture from two noble metals (Au and Ag)^77^. According to the results in Table 3, our prepared catalysts are more effective and cheaper. 100% conversion of the 4-NP solution over our catalysts was achieved in a short time with high rate constant (k) values (8.61 × 10^–3^ s^−1^ and 18.3 × 10^–3^ s^−1^) at room temperature and high efficiency using a low catalyst loading and high reactant concentration (Table 3). Our catalysts are cheap materials compared to other catalysts that contain noble metals and expensive supports such as dendrimers^74^ and graphene oxide^75^.

**Table 3:** Comparison between different catalysts that were used for the reduction of 4-NP using NaBH_4_.

Entry	Catalyst	Conditions	Efficiency (%)	Rate (s^−1^)	Refs
1	Cu_x_O@C	Cat. (2 mg), 4-NP (16 μmol), NaBH4 (1 mol)	95	4.8 × 10^–3^	^72^
2	Pd@MIL-100 (Fe)	Cat. (0.525 mg), NaBH_4_ (54 mg), 4-NP (94 mg/L)	100	6.5 × 10^–3^	^76^
3	CoO_x_/CN	Cat. (2 mg), NaBH_4_ (10 mmol), 4-NP (8 mmol)	95	4.2 × 10^–3^	^73^
4	Ag@GO	Cat. (1 mg), 4-NP (0.1 mM), NaBH_4_ (100 mM)	100	7.5 × 10^–4^	^75^
5	Au_x_Ag_1-x_	Cat. (0.2 mM), 4-NP (0.1 M), NaBH_4_ (0.25 mM)	95	1.9 × 10^–1^	^77^
6	Ag–PPDNCs	Cat. (0.44 mM), 4-NP (12.75 mM), NaBH_4_ (0.2 M)	95	7.2 × 10^–3^	^74^
7	Cu/UiO-66	Cat. (50 mg), 4-NP (50 ml, 2 mM), NaBH_4_ (1.25 ml, 2 M)	100	8.61 × 10^–3^	This work
8	CuO/ZrO_2_-SO_3_H@C	Cat. (50 mg), 4-NP (50 ml, 2 mM), NaBH_4_ (1.25 ml, 2 M)	100	18.3 × 10^–3^	This work

## Conclusions

The missing-linker defects in the UiO-66 were exploited to incorporate copper clusters inside the MOF pores through covalent bonding with the Zr_6_O_8_ nodes via Cu–O–Zr bridges. The received catalyst (Cu/UiO-66) was used to prepare CuO/ZrO_2_@C nanocomposite via carbonization at 600 °C for 3 h in air. The acidity properties of the nanocomposite were enhanced by doping with sulfuric acid. The CuO/ZrO_2_-SO_3_H@C nanocomposite displayed a complete conversion of the 4-NP into 4-AP within only 2 min of stirring at room temperature with a high reduction rate of 18.3 × 10^–3^ s^−1^. A combination between the Lewis and Brønsted acid sites on the catalyst's surface is the reason for its high catalytic activity. Moreover, the carbon sheets act as a protective agent for the prepared mixed oxides. As a result, the prepared catalysts can be reused for several cycles without significant loss of catalytic activity. Therefore, the catalysts have a high potential for the reduction of nitro compounds under mild reaction conditions.

## Supplementary Information

Supplementary Information.

## Data Availability

All data generated or analyzed during this study are included in this published article (and its Supplementary Information files).
