# Supplementary material for: In-situ preparation of sulfonated carbonaceous copper oxide-zirconia nanocomposite as a novel and recyclable solid acid catalyst for reduction of 4-nitrophenol
Source: Sci Rep. 2023 Jun 22;13:10123. doi: 10.1038/s41598-023-36627-x (PMC10287755; doi:10.1038/s41598-023-36627-x)
Supplement: Supplementary file 1 — Supplementary Information. [file 41598_2023_36627_MOESM1_ESM.docx]

**Supplementary Information**

**In-situ preparation of sulfonated carbonaceous copper oxide-zirconia nanocomposite as a novel and recyclable solid acid catalyst for reduction of 4-nitrophenol**

**Mostafa Farrag***

Chemistry Department, Faculty of Science, Assiut University, 71515 Assiut, Egypt

*****[**mostafafarrag@aun.edu.eg**](mailto:mostafafarrag@aun.edu.eg)

**Instrumentation and characterization**

Adsorption–desorption isotherms of nitrogen (−196 ^o^C) are obtained using a Quantachrome (Nova 3200 series) multi-gas adsorption apparatus. Prior to analysis, the samples were outgases at 150 ^o^C for 12 hrs. Specific surface areas are calculated from these isotherms by applying the BET equation. S_t_ Values are calculated using the V_a–t_ plots of de Bore. For HR-TEM measurements, solutions with a concentration of 1−2 mg/mL are prepared by suspension the samples in methanol. Droplets of these samples solutions are casted onto carbon-coated copper grids. The solvent is then allowed to evaporate slowly. TEM images for the prepared clusters were obtained with a JEOL JEM 2010 with LaB6-Cathode electron microscope operating at an acceleration voltage of 200 kV. The images are then analyzed by using Image J software (version 1.44). Powder X-ray diffraction (XRD) was performed on a BRUKER-D8 ADVANCE X-ray powder diffractometer, model pw 2013/00. Ni-filtered Cu Kα with a wavelength of λ = 1.541838 Å was used as a constant source of radiation. The generator was operated at 35 kV and 20 mA, and diffractometer at 50 diverting and receiving slits and a scan rate of 20 mm/min. Fine powder samples were loaded on a quartz plate holder by spreading the powders as a smooth thin layer on the plate. For all diffractograms, the following settings were used: scan range 2–80^◦^ (2θ), scan step 0.04^◦^. The elemental composition of the prepared catalysts was determined using the energy dispersive X-ray spectroscopy (EDS) and elemental mapping (EDAX Element made in USA). The surface electronic states are investigated using XPS K-ALPHA (Themo Fisher Scientific, USA) with monochromatic X-ray Al K-alpha radiation -10 to 1350 e.v spot size 400 micro m at pressure 10^-9^ mbar with full-spectrum pass energy 200 e.v and narrow-spectrum 50 e.v. The XPS data are calibrated internally by fixing the binding energy (BE) of the C1s peak at 284.6 eV. The characteristic absorption peaks of 4-nitrophenol and 4-aminophenol were investigated also by UV-vis spectroscopy. The spectra of all the solutions were recorded at ambient temperature from 200 to 900 nm with a double-beam spectrophotometer (Evolution 300). The used gas chromatography (GC) is Thermo Scientific, Trace GC ultra-using capillary column TG-5MS (5% Phenyl Methyl polysiloxane), length 30 m, internal diameter 0.25 mm, and film thickness 0.25 µm. The initial temperature of column is 50 ^o^C and the final temperature is 300 ^o^C, the rate of heating is 10 ^o^C/min. The injector temperature is 350 ^o^C and the detector temperature is 350 ^o^C, flame ionization detector is the used detector.





**Figure S1.** The change in the concentration of 4-NP with time during the reduction reaction over the prepared catalysts, (the reaction conditions are 50 ml of 4-NP (2 mM), 50 mg of the catalyst, 1.25 ml of NaBH_4_ (2 M), and 1000 rpm).


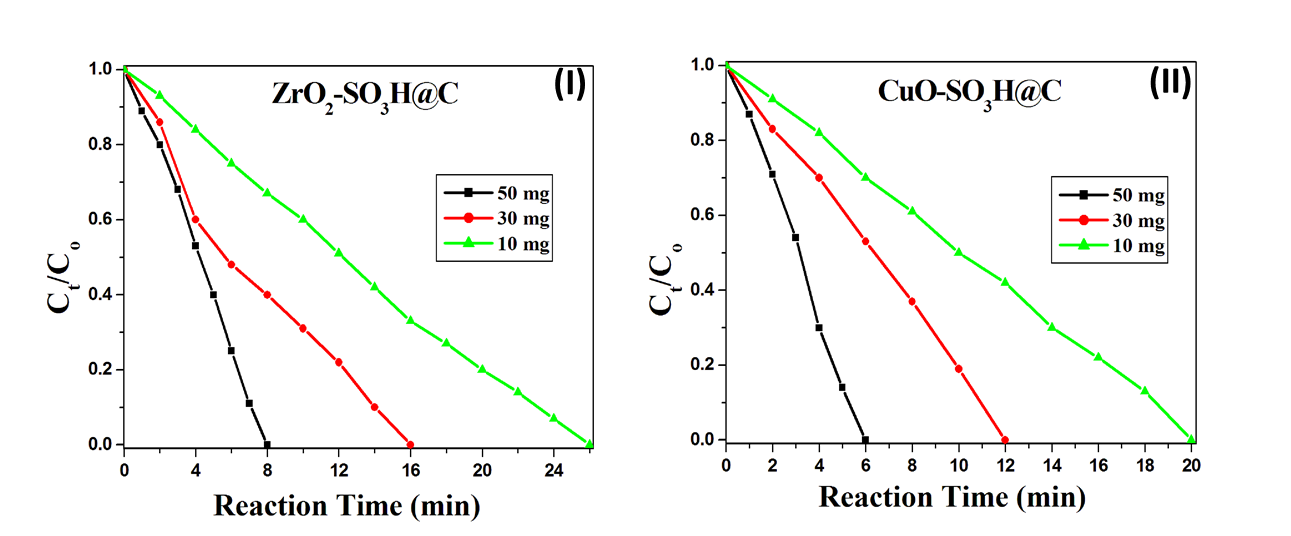


**Figure S2.** The effect of catalyst weight on the reduction of 4-NP over the ZrO_2_-SO_3_H@C (**I**) and CuO-SO_3_H@C (**II**). The experimental conditions were kept constant, but the catalyst weight was changed only (50, 30, and 10 mg).

**Figure S3.**The mechanism for the reduction of 4-nitrophenol to 4-aminophenol.
